# Multi-Scale Attention-Guided Non-Local Network for HDR Image Reconstruction

**DOI:** 10.3390/s22187044

**Published:** 2022-09-17

**Authors:** Howoon Yoon, S. M. Nadim Uddin, Yong Ju Jung

**Affiliations:** School of Computing, Gachon University, Seongnam 13120, Korea

**Keywords:** high-dynamic-range imaging, spatial attention, non-local means, deep learning

## Abstract

High-dynamic-range (HDR) image reconstruction methods are designed to fuse multiple Low-dynamic-range (LDR) images captured with different exposure values into a single HDR image. Recent CNN-based methods mostly perform local attention- or alignment-based fusion of multiple LDR images to create HDR contents. Depending on a single attention mechanism or alignment causes failure in compensating ghosting artifacts, which can arise in the synthesized HDR images due to the motion of objects or camera movement across different LDR image inputs. In this study, we propose a multi-scale attention-guided non-local network called MSANLnet for efficient HDR image reconstruction. To mitigate the ghosting artifacts, the proposed MSANLnet performs implicit alignment of LDR image features with multi-scale spatial attention modules and then reconstructs pixel intensity values using long-range dependencies through non-local means-based fusion. These modules adaptively select useful information that is not damaged by an object’s movement or unfavorable lighting conditions for image pixel fusion. Quantitative evaluations against several current state-of-the-art methods show that the proposed approach achieves higher performance than the existing methods. Moreover, comparative visual results show the effectiveness of the proposed method in restoring saturated information from original input images and mitigating ghosting artifacts caused by large movement of objects. Ablation studies show the effectiveness of the proposed method, architectural choices, and modules for efficient HDR reconstruction.

## 1. Introduction

High-dynamic-range (HDR) imaging techniques extend the range of brightness in an image such that a photograph taken by a camera becomes as similar as possible to the scene as it would be observed by the naked eye. HDR images have been used in a wide range of applications such as photography, film, and games [1,2] as they contain more information from a given scene and can provide a better visual experience. However, because camera image sensors generally have a narrow dynamic range, capturing scenes with more widely varying ranges of brightness using a single exposure value is considered relatively difficult [3,4]. Recent advances in imaging technology have enabled the acquisition of HDR images with only a single-sensor camera, but these special cameras are often very expensive for consumers in general. Therefore, the primary method used to obtain HDR imaging is to continuously photograph and overlap multiple images with a low dynamic range (LDR) with different exposure values and then select or fuse the best photo pixels or segments to reconstruct a single HDR image [5,6,7].

Traditional methods mostly depend on rejection-based approaches [8,9,10,11,12,13,14,15,16,17], rigid and non-rigid registration-based approaches [18,19,20,21,22,23] and patch-based optimization methods [24,25] for reconstructing HDR images. Rejection-based HDR reconstruction methods produce high-quality HDR contents for static scenes with LDR contents for moving regions, thus limiting their scope for static scenes mostly. Registration-based traditional approaches work plausibly well with static scenes and small movements, however, fail in complex and dynamic scenes with large motions and occluded pixels. Patch-based optimization methods, though capable of high-quality HDR image reconstruction, suffer from high computational complexity and often fail to produce plausible HDR images in run-time.

To alleviate the HDR reconstruction problem, recently, various deep learning methods based on convolutional neural network (CNN) have been utilized to perform multi-exposure-based multi-frame HDR imaging [26,27,28,29,30,31]. CNN-based methods have shown significant promise in producing HDR contents with fewer LDR images (i.e., typically three LDR images) for comparatively complex scenes. Early studies focused on pre-processing methods to align LDR images beforehand [32,33,34,35] using optical flow [22,23,28,29] or homography [29,34,36,37], and then input the data to convolutional neural network (CNN) models. However, although properly coping with small movements of objects in this way has been shown to be possible, the performance of these methods is limited by the accuracy of the alignment. Moreover, these methods often fail in scenes with large motions and saturated regions.

**Figure 1:**
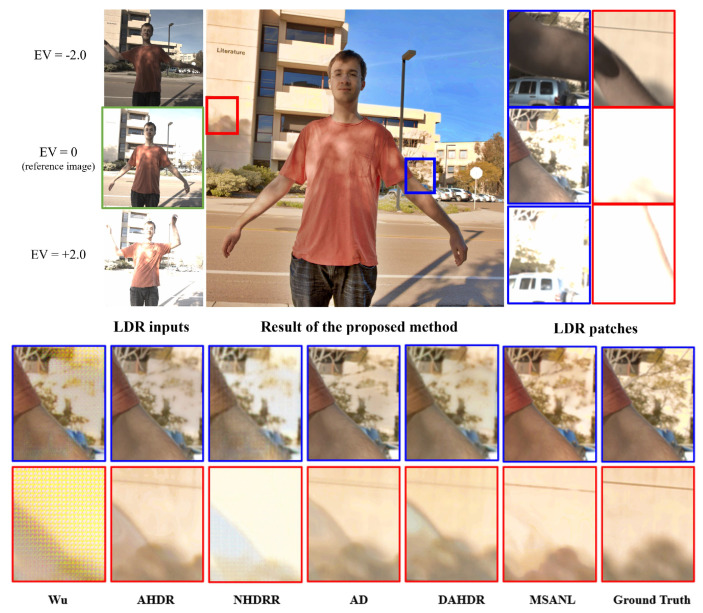
Visual comparison of HDR image restoration results obtained using the proposed method and state-of-the-art methods. From left, Wu [28], AHDR [26], AD [27], NHDRR [30], DAHDR [39], Proposed MSANLnet, and Ground Truth.

Recent CNN models are focused on performing feature-level attention-based implicit alignment mechanisms that use contextual information from the LDR images to adaptively select pixels/regions for efficient fusion [26,27,30,38]. These methods enabled the movement of an object to be properly processed while maintaining important information for each input image. The key challenge of such a multi-frame HDR imaging approach is to properly handle the “ghosting artifacts” caused by inappropriate image fusion due to objects moving within a scene or camera instability. For example, as shown in Figure 1, if images captured continuously at different times are improperly processed and fused, different objects appear to overlap in the resulting image, i.e., ghosting artifacts, due to the motion of the objects.

In this study, we propose a deep neural network, called MSANLnet, that uses the multi-scale attention mechanism and non-local means technique to effectively alleviate the ghosting artifact problem in HDR image reconstruction. The proposed MSANLnet has two distinctive parts—(a) multi-scale attention for object motion and saturation mitigation, and (b) non-local means-based fusion. Existing spatial-wise attention only captures important regions on a single scale, which is not effective for distinguishing both object movements and saturated regions in the case of producing HDR contents. In the proposed method, LDR features are aligned based on multi-scale spatial attention modules. During this process, spatial attention is performed for each scale of features to expand the size of the receptive field and progressively correct and fuse important information, such as large movements and saturation, which is found to be effective in producing high-quality HDR content. Again, the non-local means module looks at the whole image and only fuses the correlated global contents with the reference image. Hence, the multi-scale attention mechanism can effectively capture the local movements of the objects and the non-local means-based fusion can reduce ghosting artifacts on a global level by looking at the whole image, which effectively mitigates ghosting artifacts for large object motions and global shifts such as camera translation.

This two-stage design effectively selects different exposure values for each input frame and useful information that is not altered by motion and then fuses images to minimize locality to reduce noise and mitigate the ghosting artifact problem. We validated that the proposed method performs better than the existing methods quantitatively and qualitatively through the experiments on a publicly available dataset that has been mainly used for HDR image restoration. We summarize our contributions as follows.

- We propose MSANLnet, a multi-scale attention-guided non-local network for HDR image reconstruction, that extracts important features from the LDR features using the multi-scale spatial attention and adaptively fuses the contextual features to obtain HDR images.
- We show that the multi-scale spatial attention, combined with the non-local means-based fusion, can effectively alleviate the “ghosting artifact” and produce aesthetic HDR images.
- Our proposed method outperforms the existing methods in both qualitative and quantitative measures, validating the efficacy of the attention modules, non-local means-based fusion, and architectural choices.

The remainder of this study is organized as follows. Related work is discussed in Section 2. The proposed MSANLnet is presented along with detailed descriptions of each module in Section 3. The experimental setup and results are presented in Section 4. Finally, Section 5 presents our conclusions and suggests some possible avenues for further research.

## 2. Related Work

First proposed by Madden [40] and Mann [41] and popularized by Debevec and Malik [42] for digital imaging, multi-exposure-fusion-based HDR reconstruction methods can be classified into two major classes—(i) traditional approaches, and (ii) deep learning-based approaches. For brevity, we restrict our related work only to multi-exposure-fusion-based approaches.

Rejection-based methods try to find the pixels/regions with motions and select only pixels/regions using a reference image or the static contents of the LDR images [5,8,12], i.e., these methods reject the moving contents. Rejection mechanisms are typically implemented by performing patch-wise comparison [9], illumination consistency and linear relationships among pixels of the LDR images [16,43,44], thresholding [5,11,12], background probability map [45], super-pixel comparison [15] etc. Such approaches can produce fast results, however, are limited to producing LDR contents in the dynamic regions.

Alignment-based traditional methods typically select one reference exposure LDR image and register the remaining LDR images either using rigid registration mechanisms (e.g., SIFT-based [18], SURF-based [10], Median Threshold Bitmap-based [19] methods, etc.) or non-rigid registration mechanisms (e.g., optical flow-based methods [20,22,23,46]). These methods can produce plausible results for static scenes and small motions, however, perform poorly in recovering saturated pixels.

Patch-based optimization methods typically synthesize LDR patches based on the structure reference patches by finding dense correspondence among the LDR images and fuse the synthesized LDR images to produce HDR contents [24,25,47]. These methods can produce high-quality HDR content but suffer from computational complexity. Though most of the traditional methods produce plausible HDR contents for static scenes, they suffer from poor synthesis/misalignment of LDR contents in dynamic scenes with large motions and recovering saturated pixels/regions from the input LDR images.

Deep learning-based methods are capable of synthesizing novel HDR contents based on the input LDR images, making them suitable for complex dynamic scenes. Early methods employed CNN-based methods, e.g., optical flow estimation [28,29], multi-scale feature extraction [38], etc., to compensate ghosting artifacts. Subsequently, attention-based alignment methods are used to remove ghosting artifacts and recover saturated regions [26,27,39,48].

Deep learning-based multi-scale attention mechanisms have also shown significant promise in various image reconstruction tasks, e.g., semantic image synthesis [49,50], generative image synthesis [51,52,53], image inpainting [54,55,56,57], segmentation [58,59,60,61,62,63,64], super-resolution [65,66,67,68,69,70,71,72,73,74], image enhancement [75], etc. Though several HDR approaches utilize multi-scale feature fusion [26,38,39,76,77,78,79,80,81], the removal of object motion and the mitigation of saturation using multi-scale attention mechanisms remain an under-explored research area.

Recently, generative adversarial networks (GANs) have been employed to supervise the HDR reconstruction process, which can effectively correct large motions with higher efficiency [82]. Despite the success of deep learning-based approaches, removing ghosting artifacts in HDR images for large motions is still an active area of research.

## 3. Methods

### 3.1. Overview of MSANLnet

The MSANLnet takes three input LDR tensors as inputs. Each tensor consists of an LDR image and the tone-mapped HDR image of the corresponding LDR image. First, the three LDR images in the input LDR set (i.e., $L=\{L_{1},L_{2},L_{3}\}$, where $L_{i=\{1,2,3\}}\;\in R^{H\times W\times 3}$) are arranged according to their exposure lengths $t_{i}$, i.e., short exposure ($L_{1}$), intermediate exposure ($L_{2}$), and long exposure length ($L_{3}$). Then, a mapping process is performed on the input LDR image set L to convert them into the input HDR image set, $H=\{H_{1},H_{2},H_{3}\}$, $H_{i=\{1,2,3\}}\;\in R^{H\times W\times 3}$. Here, the gamma correction (γ=2.2, following [26]) is used for the mapping process as follows:

$$H_{i}=\frac{{\left(L_{i}\right)}^{\gamma }}{t_{i}};i=1,2,3,$$

Then, a pair of input tensors $X_{i}$ for each $L_{i}$ is obtained by concatenating the gamma-corrected $H_{i}$ and the corresponding $L_{i}$ in the channel dimension, i.e., $X_{i}=\left\{H_{i},L_{i}\right\}$, where $X_{i}\;\in R^{H\times W\times 6}$. Among these tensors, $X_{2}$ with the intermediate exposure length is selected as a reference tensor. The set of input tensors X is then fed to MSANLnet to produce an HDR image.

We define the process of generating the final HDR image $H_{f}$ by fusing the three tensors, $X=\{X_{1},X_{2},X_{3}\}$, as follows,

(1) $$H_{f}=f\left(X_{1},X_{2},X_{3};\theta \right),$$

where *f* represents the proposed MSANLnet and θ is a parameter of the corresponding network.

**Figure 2:**
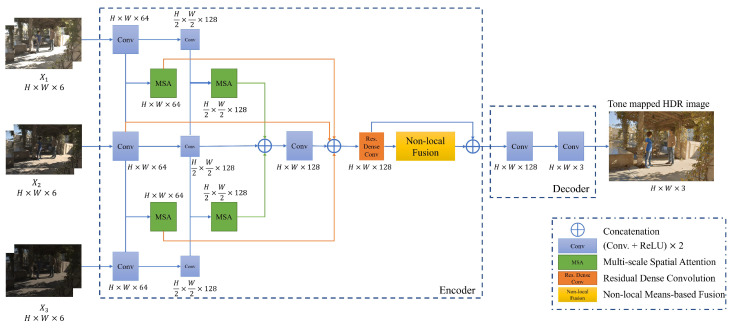
Overview of the proposed MSANLnet for HDR image reconstruction.

The proposed MSANLnet model uses a CNN-based encoder-decoder structure and is largely composed of multi-scale spatial attention modules and non-local means-based fusion modules. Figure 2 shows the overview of the proposed network. As shown in Figure 2, the encoder extracts spatial attention features based on the scale of LDR tensor set $X_{i}$ through the multi-scale spatial attention module (please refer to Section 3.2 and Supplementary Materials). The multi-scale spatial attention features contribute to compensating motions and recovering feature values from the saturated regions adaptively. The decoder uses a transposed convolution block to restore the reduced resolution to the original resolution while concatenating spatial attention features and reference image features. Finally, the final HDR image is output through a non-local means-based fusion mechanism (please refer to Section 3.3 and Supplementary Materials) which considers the global relationships among feature values, thus effectively removing ghosting artifacts for larger motions during the HDR image reconstruction.

### 3.2. Multi-Scale Spatial Attention Module

The proposed approach is designed to effectively identify areas to be used in images with long exposure values and images with short exposure values. Specifically, a multi-scale attention mechanism is devised in the encoder to implicitly align the two images with the reference image, as shown in Figure 2.

First, three 6-channel input tensors $X_{1}$, $X_{2}$, $X_{3}$ are encoded in spatial multi-scales, respectively, via a convolutional layer to extract features. During this process, max pooling is applied to reduce the spatial size of the feature map extracted at each step by half from the previous step. Thus, the receptive field is increased to capture a larger foreground movement in the multi-scale attention module. Note that when we decrease the spatial size of a feature map by half, we increase its number of channels by double. For example, in our experiment, we used 64 channels for the first scale and 128 channels for the second scale.

**Figure 3:**
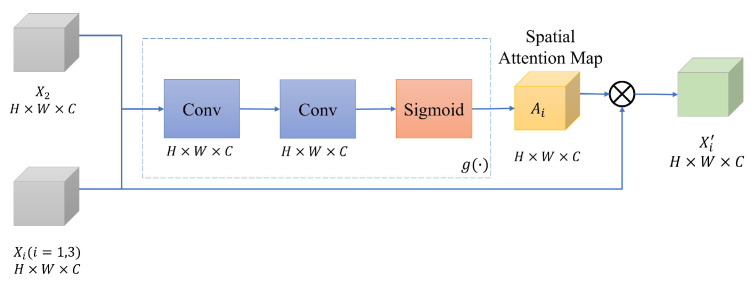
Overview of a spatial attention block.

As shown in Figure 2, a spatial attention block is executed for each scale to extract the spatial attention maps of the non-reference images. A spatial attention block is shown in Figure 3. In the spatial attention block, the input is the concatenation of the non-reference image features, $X_{i};i\neq 2$, and the reference image $X_{2}$ features. Then, the spatial attention map is extracted at each scale *s* as follows,

(2) $${A_{i}}^{\left(s\right)}=g\left({X_{i}}^{\left(s\right)},{X_{2}}^{\left(s\right)}\right);i=1,3;s=1,2,\ldots ,N,$$

where *s* denotes the number of spatial scales and g() is the spatial attention map extractor. The attention map extractor consists of two consecutive convolutions and sigmoid function, as used in a previous study [26]. Note that, in our experiment, we used *N*=2 (i.e., two scales as depicted in Figure 2). An element of the spatial attention map $A_{i}$ is in a range of [0−1] and the spatial size of the generated $A_{i}^{\left(s\right)}$ is equal to $X_{i}^{\left(s\right)}$.

Finally, an element-wise multiplication between the input feature ${X_{i}}^{\left(s\right)}$ and the extracted ${A_{i}}^{\left(s\right)}$ is performed to generate the final spatial attention feature ${X_{i}^{\prime }}^{\left(s\right)}$ of the images.

(3) $${X_{i}^{\prime }}^{\left(s\right)}={A_{i}}^{\left(s\right)}\cdot {X_{i}}^{\left(s\right)};i=1,3;s=1,2,\ldots ,N.$$

**Figure 4:**
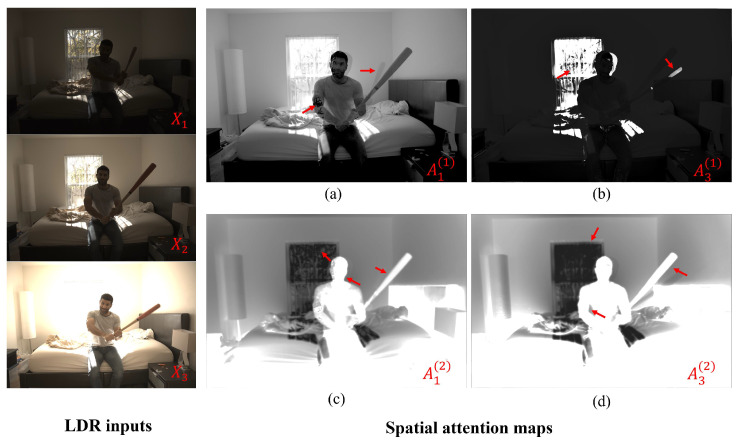
Visualization of spatial attention maps. The attention maps for the first scale are shown in (**a**,**b**). The attention maps for the second scale are shown in (**c**,**d**).

As seen in Figure 4, two spatial attention maps focus on important regions for implicit alignment of the input LDR images to the reference image. As the network is provided with explicit multi-scale contextual information, it can compensate for large foreground motions and can effectively mitigate the ghosting artifacts. Moreover, as the spatial attention maps represent the differences among the LDR images with varying exposures, the network implicitly learns to recover saturated pixel values.

### 3.3. Non-Local Means-Based Fusion

In some local areas of the image feature, sufficient information is not available due to occlusion or saturation caused by the movement of objects [26]. Therefore, in image fusion networks, useful information must be extracted into a large receptive field. However, a single CNN convolution filter can be used in only a limited area of the receptive field. Previous studies have shown the importance of short and long-range dependencies among the pixel/feature values for various computer vision tasks [54,83,84,85,86]. Non-local means is a method of calculating long-range dependencies and restoring pixels through weighted averages based on these dependencies, intuitively beneficial for removing ghosting artifacts. To this end, we applied a non-local block to image fusion so that global information is utilized and locality is reduced to effectively alleviate ghosting artifacts.

**Figure 5:**
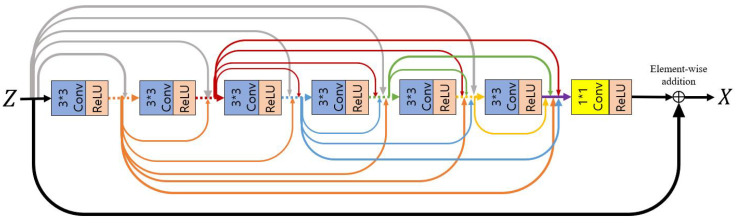
A residual dense convolutional block is composed of dense concatenations based on skip-connections between a series of convolutional blocks consisting of convolutions and ReLU activation functions.

First, we utilize the residual dense convolutional block [87] before applying the non-local block, such that the feature Z recovered to the original size through the decoding process learns sufficient local information. By concatenating all layers, useful local information is adaptively extracted through the features of the previous layer and the features of the current layer. The extracted *X* enters a non-local block input and outputs *Y* of the same size, H×W×C, as a result. Figure 5 illustrates the structure of a residual dense convolutional block composed of three convolutional blocks.

**Figure 6:**
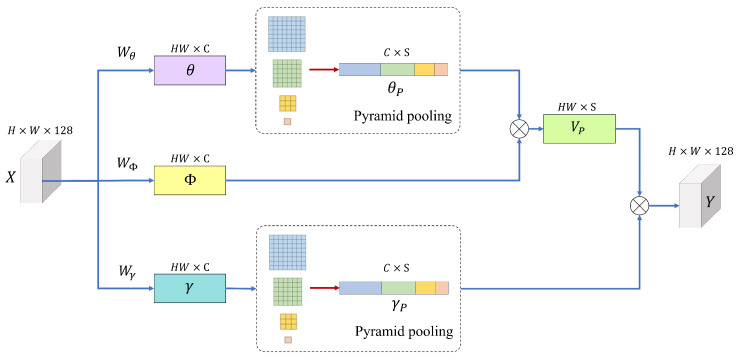
Structure of the asymmetric pyramidal non-local block with spatial pyramid pooling [88].

For the non-local based fusion, we adopt the asymmetric pyramid non-local block (APNB) [88] to construct the non-local blocks. In APNB, spatial pyramid pooling is applied to a standard non-local block to reduce computation by sampling part of the feature map based on meaningful global information. Figure 6 shows the structure of an asymmetric pyramidal non-local block, including spatial pyramid pooling.

First, the input feature map X is converted to different embeddings, Φ, θ, and γ, respectively, through three 1×1 convolutions, $W_{\Phi }$, $W_{\theta }$ and $W_{\gamma }$.

(4) $$\Phi =W_{\Phi }\left(X\right);\theta =W_{\theta }\left(X\right);\gamma =W_{\gamma }\left(X\right).$$

The spatial pyramid pooling module is composed of four pooling layers that derive the results of different sizes in parallel. Such a pyramid pooling mechanism improves the expressive power of global features as previous studies have shown the effectiveness of the global and multi-scale representations for capturing scene semantics. The sampling process through pyramid pooling $P_{\theta }$ and $P_{\gamma }$ are represented by the following equation.

(5) $$\theta_{P}=P_{\theta };\gamma_{P}=P_{\gamma }.$$

The number of spatially sampled anchor points *S* can be expressed as,

(6) $$S=110=\sum_{n\in 1,3,6,8}n^{2},$$

where *S* denotes the number of sampled anchor points and *n* denotes the width of the output features processed through the pooling layer. Thereafter, based on the standard non-local block method [89], the pseudo-matrix $V_{P}$ of $\theta_{P}$ and Φ is calculated as follows,

(7) $$V_{P}=\Phi^{T}\times \theta_{P}.$$

Instance normalization is then applied to $V_{P}$ to generate a normalized pseudo-matrix $V_{P}$, and the Softmax function of the self-attention mechanism is used to derive the attention result as follows,

(8) $$Z=V_{P}\times \gamma_{P}^{T}.$$

The final output of the asymmetric pyramidal non-local block is obtained as follows,

(9) $$Y=cat(W_{o}\left(Z\right),X),$$

where $W_{o}$ is composed of 1×1 convolution and can be learned by weighting the parts where parameters are important in the non-local operation. Subsequently, the network generates a final 3-channel HDR image through a convolutional layer.

## 4. Experimental Results

### 4.1. Dataset, Objective Function, Implementation Details, and Evaluation Design

#### 4.1.1. Dataset

We used an open dataset [28] for HDR performance comparison to train and evaluate the proposed method. Specifically, out of a total of 89 image sets, 74 image sets were used as a training set, 5 were used as a validation set, and the remaining 10 were used as a testing set. Each image set comprised three LDR images and one HDR ground-truth image. Here, three LDR images were captured using exposure bias {−2,0,+2} or {−3,0,+3}. As in an existing study on HDR [26], in the training stage, a total of 1775 image patches were used to perform training by cropping LDR images with a size of 1000×1500 into 256×256 patch sizes and using them with a stride of 128 through data augmentation to mitigate over-fitting.

#### 4.1.2. Objective Function

As proposed in a previous work [28], HDR images were mainly presented after being tone-mapped, and thus, it was more effective to optimize the network in the tone-mapping domain than in the HDR domain. A tone-mapping process using a μ-law was performed on the HDR image H and ground-truth image GT derived in this study as follows.

(10) $$T\left(X\right)=\frac{log(1+\mu X)}{log(1+\mu )},$$

where μ is a parameter indicating the degree of compression, and T(X) denotes a tone-mapped image. We set μ=5000 [28], and the range of the resulting T(X) is [0,1]. Subsequently, to minimize the error value between T(H) and T(GT) by applying per-pixel $L_{1}$-Loss, the network was trained after defining the loss function L as follows,

(11) $$L={\left|T(\mathrm{GT})-T(H)\right|}_{1}.$$

#### 4.1.3. Implementation Details

In the training stage of the proposed method, the number of epochs and batch size were set to 200 and 4, respectively. The learning rate was initially set to 0.0001, and then the learning rate was reduced by multiplying by 0.1 at 100 epochs. We used the Adam optimizer [90], and both learning and evaluation were implemented using the Pytorch framework [91]. Our experiments are performed on a single NVIDIA 2080Ti GPU. The number of model parameters is 2.8 million (2,772,707) and the average inference time per image is 0.0181 s.

#### 4.1.4. Evaluation Design

To evaluate the efficiency of the proposed method, we compared it with five state-of-the-art methods, both in qualitative and quantitative manner. Specifically, we compared our method with AHDR [26], Wu [28], AD [27], NHDRR [30], and DAHDR [39]. AHDR [26] introduced spatial attention modules in HDR imaging to guide the merging according to the reference image. AD [27] used spatial attention module for feature-level attention and a multi-scale alignment module (i.e., PCD alignment [92]) to align the images in the feature-level. DAHDR [39] exploits both spatial attention and feature channel attention to achieve ghost-free merging. NHDRR [30] is based on U-Net-based architecture with non-local means to produce HDR images. All of the selected models are deep learning-based methods for generating an HDR image using multiple LDR images. In addition, all of these models used LDR images with large motions as training datasets, which were considered appropriate to objectively compare performance with our method. All models were trained in the same way and in the same environment as the proposed approach for a fair comparison.

### 4.2. Quantitative Evaluation

To quantitatively evaluate the performance of the model, we measured the peak signal-to-noise ratio (PSNR) and structural similarity index map (SSIM) on the testing set. PSNR is used to evaluate the impact of lost information on the quality of a generated or compressed image and represents the power of received noise with respect to the maximum power of the received signal. SSIM is a method designed to evaluate human visual quality differences rather than numerical loss and evaluates quality by comparing luminance, contrast, and structure values that make up images, rather than only performing comparisons between pixels. We used the PSNR-*l* and PSNR-μ indicators, which measure PSNR in linear and tone-mapped domains, respectively. For the SSIM, we used values measured in tone-mapped domains.

Table 1 shows the average value of the quantitative indicators, and the larger values correspond to the higher quality. The proposed model achieved higher values than other models in all three quantitative performance indicators. The proposed method achieved better performance in terms of PSNR-*l*, PSNR-μ, and SSIM measurements compared to AHDR [26], Wu [28], AD [27], NHDRR [30], and DAHDR [39]. This occurred because the proposed method can restore details and effectively eliminate ghosting artifacts through non-local means-based fusion modules. NHDRR [30] also merges images using non-local means modules, but MSANLnet, which extracts features using scale-specific spatial attention, exhibited numerically better performance.

### 4.3. Qualitative Evaluation

**Figure 7:**
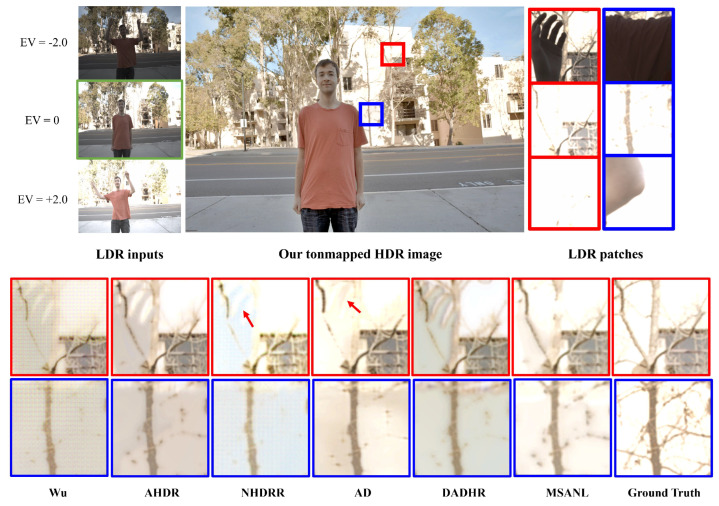
Qualitative comparison of HDR result images of the proposed method (MSANLnet) and state-of-the-art models. From left, Wu [28], AHDR [26], AD [27], NHDRR [30], DAHDR [39], Proposed MSANLnet, and Ground Truth.

**Figure 8:**
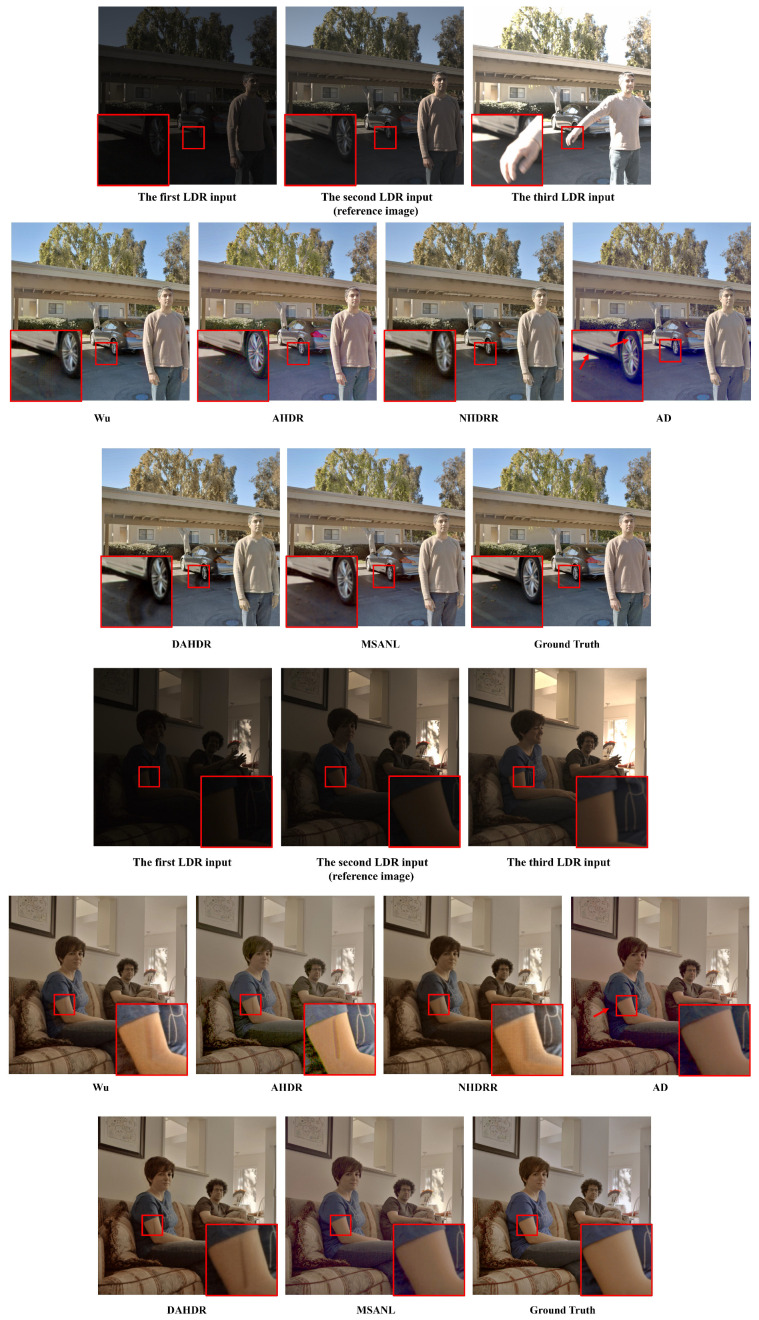
Visual comparison of HDR images restored from the dataset provided in [28]. From left, Wu [28], AHDR [26], NHDRR [30], AD [27], DAHDR [39], Proposed MSANLnet, and Ground Truth.

Figure 7 and Figure 8 visually compare the results of HDR image generation via the proposed method and existing state-of-the-art models [26,27,28,30,39]. In particular, Figure 7 qualitatively compares the experimental results of images with saturated backgrounds and large foreground movements. Input images referenced by the network by exposure value are shown in Figure 7a, and the tone-mapped HDR result images produced by the proposed method are shown in Figure 7b. The corresponding testing set image had a saturated background area and large foreground movements. Areas with large movements and saturation levels were cut into patches as shown in Figure 7b,c, respectively. The details of the background were obscured by the movement of an object in an image with a small exposure length, and the information was lost even in an image with long or medium exposure lengths. Because the image was saturated, learning models were likely to use artifacts and distorted information from the first image with the background occluded for HDR imaging, as seen in Figure 7c.

As seen in Figure 7, Wu [28] produced an excessively smooth image and failed to restore the details of the saturated area and removed ghosting artifacts. Although AHDR [26] selectively incorporated useful areas through spatial attention modules to restore obscured or saturated details, they still exhibited ghosting artifacts. NHDRR [30] removed ghosting artifacts to some extent, but output some blurring artifacts and missed the details of many areas. AD [27] uses PCD alignment modules [92], which reduce ghosting artifacts to some extent. However, the over-exposed area was not fully recovered. In DAHDR [39], the afterimage of the finger remains intact, which is not effective in removing ghosting artifacts. Furthermore, it can be seen that the hidden area cannot be restored as well. The results of the proposed MSANLnet expressed more details compared to the existing methods, while also eliminating ghosting artifacts, as may be observed from the figures. The resulting images demonstrate that the proposed MSANLnet expresses more details of saturated regions through the scale-specific spatial attention modules, and effectively reduces ghosting artifacts through non-local means-based fusion.

Figure 8 also shows a comparison of images with motion and supersaturation regions in the testing set. The proposed MSANLnet restored details more clearly compared to other models, and also removed visual artifacts to produce higher-quality HDR images.

### 4.4. Ablation Study

#### 4.4.1. Ablation on the Network Structure

We performed an ablation study of the proposed network structure and analyzed the results. We compared the proposed MSANLnet with the following variant models to identify the importance of the individual components. Table 2 shows the quantitative comparison among the baselines and the proposed MSANLnet.

- ANLnet: The multi-scale attention module was removed from this version; i.e., this was a variant model using a single-scale attention module of the baseline model AHDR [26] and a non-local means-based fusion module.
- MSANet: The non-local fusion module was removed from this version. That is, this variant adopted a multi-scale attention module and dilated residual dense block (DRDB)-based fusion as used in baseline models.
- MSANLnet: The proposed MSANLnet model, which includes a multi-scale attention module and a non-local means-based fusion module.

**Figure 9:**
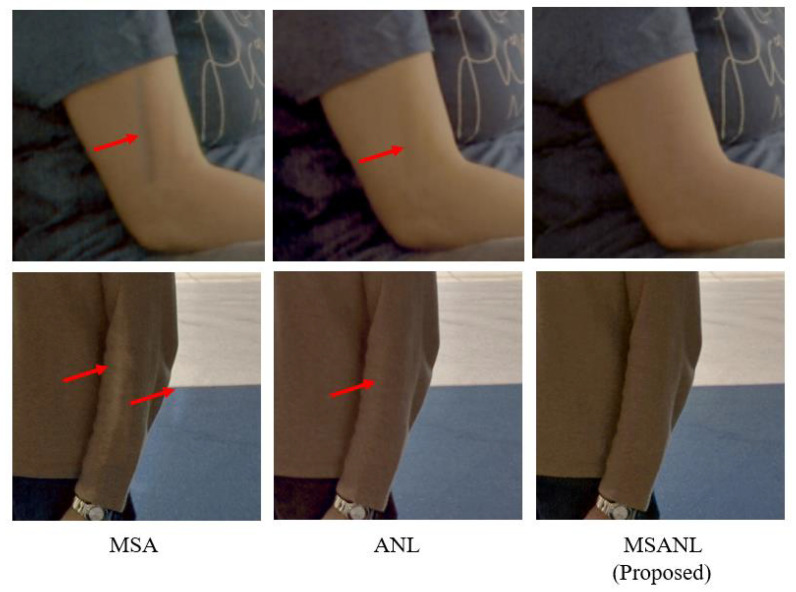
Qualitative comparison of the performance of the proposed method (MSANLnet) and its baseline variants.

As shown in Table 2, when a multi-scale attention module was used, the performance in terms of PSNR was improved by 1.41 dB compared to the use of a single-scale module. As seen in Figure 9, an afterimage of arm movement remains in MSAnet, and the difference cannot be repaired naturally, creating boundaries. However, it can be seen that the proposed model with multi-scale attention effectively eliminates such artifacts. These results confirm that expanding the receptive field helps reduce ghosting artifacts and restore details.

#### 4.4.2. Comparison of Degradation of HDR Restoration for Global Motion

**Figure 10:**
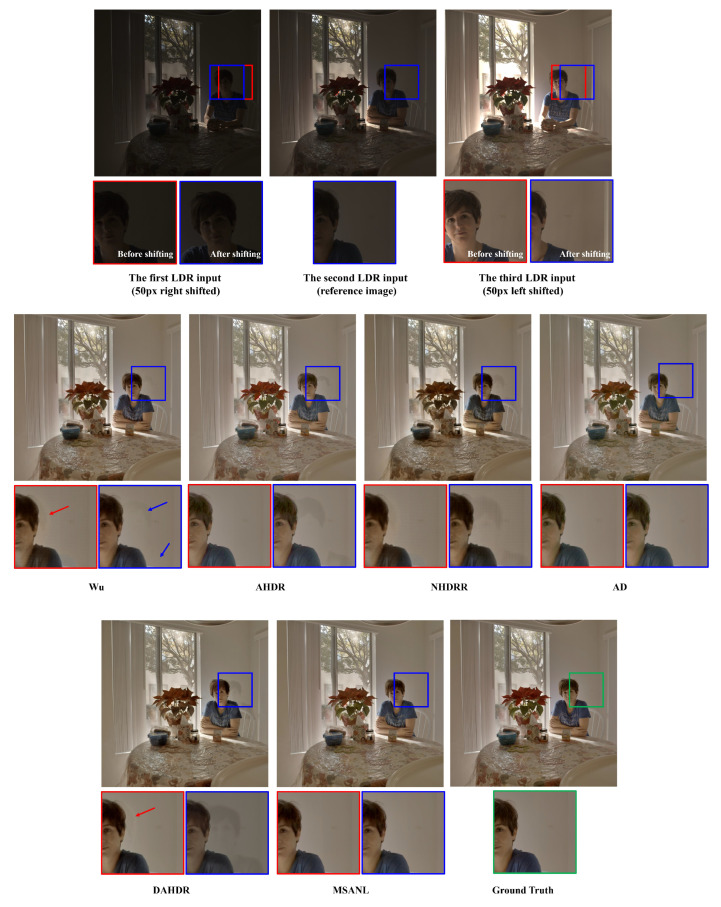
Visual comparison of degradation of HDR restoration for global motion (i.e., when applied translation). From left, Wu [28], AHDR [26], NHDRR [30], AD [27], DAHDR [39], and Proposed MSANLnet.

We have performed additional experiments with the translation applied to the input LDR images and visually compared the HDR restoration performance between the proposed and existing methods. Specifically, we globally shifted the first input LDR image to the right by 50 pixels, kept the reference LDR image (i.e., the second image) unchanged, and shifted the third input LDR image to the left by 50 pixels, to simulate the global translation operation. The visual results are summarized in Figure 10.

As seen in Figure 10, most of the existing methods fail to mitigate ghosting artifacts in the case of such global motion caused by the camera’s translation. However, our proposed method can effectively produce HDR image contents without severe quality degradation, even though we do not employ any explicit alignment mechanisms. This is because the multi-scale attention mechanism can effectively capture the local movements of the objects and the non-local means-based fusion reduces ghosting artifacts on a global level by looking at the whole image, which essentially mitigates ghosting artifacts for large object motions and global shifts such as translation.

#### 4.4.3. Generalization Ability on Different Dataset

**Figure 11:**
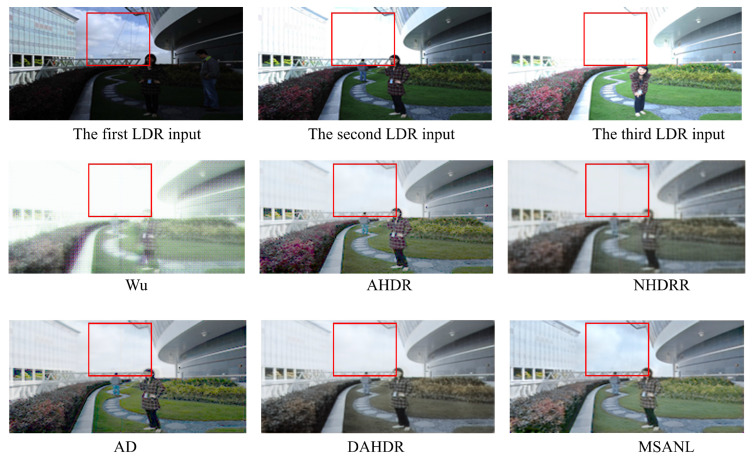
Visual comparison of additional HDR images from the dataset provided in [93]. From left, Wu [28], AHDR [26], NHDRR [30], AD [27], DAHDR [39], and Proposed MSANLnet.

We performed additional experiments on the generalization ability of the proposed method on a different test dataset. Specifically, we used test dataset provided in [93] and summarized the visual results in Figure 11. As seen in the figure, our method can generalize to unseen test datasets and performs comparatively better than the existing methods in restoring HDR contents, even though our method was not trained on the dataset.

## 5. Conclusions

In this study, we have proposed MSANLnet as a non-local network based on multi-scale attention for effective HDR image restoration. In the encoder part, implicit alignment of features is performed at various resolutions with multi-scale spatial attention modules. In the decoder part, image restoration is performed by adaptively incorporating useful information utilizing a long-range dependency with a non-local means-based fusion module. The results show that the proposed method exhibited better performance than existing deep learning methods. The results of this study have confirmed the importance and impact of the expansion of the receptive field in HDR image restoration based on CNN models. In the future, we plan to further improve the quality of HDR images by applying and developing vision transformer-based [94] modules with long-range dependencies.

## Figures and Tables

**Table 1:** Quantitative comparison of the performance of the proposed method (MSANLnet) and state-of-the-art models.

Method/Metric	PSNR-*l* ↑	PSNR-μ ↑	SSIM ↑
NHDRR [30]	33.6970	39.2461	0.9382
Wu [28]	36.4266	41.9838	0.9618
AHDR [26]	38.0948	39.5937	0.9757
AD [27]	37.9287	40.8102	0.9812
DAHDR [39]	36.5915	40.0708	0.9753
MSANL (proposed)	40.4370	42.7466	0.9821

**Table 2:** Quantitative comparison of the performance of the proposed method (MSANLnet) and its baseline variants.

Method/Metric	PSNR-*l*	PSNR-μ	SSIM
MSAnet	37.1958	39.5646	0.9699
ANLnet	39.0193	40.6810	0.9781
MSANLnet (Proposed)	40.4370	42.7466	0.9821

## Data Availability

Not applicable.

## Supplementary Materials

The following supporting information can be downloaded at: https://www.mdpi.com/article/10.3390/s22187044/s1, Table S1: Architecture of the Encoder. Table S2: Architecure of the Decoder.
