# Supplementary material for: Multi-Scale Attention-Guided Non-Local Network for HDR Image Reconstruction
Source: Sensors (Basel). 2022 Sep 17;22(18):7044. doi: 10.3390/s22187044 (PMC9503481; doi:10.3390/s22187044)
Supplement: Supplementary file 1 [file sensors-22-07044-s001.zip › sensors-1852214-supplementary.pdf]

sensors

Article

Multi-Scale Attention-Guided Non-Local Network for HDR Image Reconstruction

Howoon Yoon, S. M. Nadim Uddin and Yong Ju Jung *

School of Computing, Gachon University, Seongnam 13120, Korea
* Correspondence: yjung@gachon.ac.kr

Abstract: High-dynamic-range (HDR) image reconstruction methods are designed to fuse multiple Low-dynamic-range (LDR) images captured with different exposure values into a single HDR image. Recent CNN-based methods mostly perform local attention- or alignment-based fusion of multiple LDR images to create HDR contents. Depending on a single attention mechanism or alignment causes failure in compensating ghosting artifacts, which can arise in the synthesized HDR images due to the motion of objects or camera movement across different LDR image inputs. In this study, we propose a multi-scale attention-guided non-local network called MSANLnet for efficient HDR image reconstruction. To mitigate the ghosting artifacts, the proposed MSANLnet performs implicit alignment of LDR image features with multi-scale spatial attention modules and then reconstructs pixel intensity values using long-range dependencies through non-local means-based fusion. These modules adaptively select useful information that is not damaged by an object's movement or unfavorable lighting conditions for image pixel fusion. Quantitative evaluations against several current state-of-the-art methods show that the proposed approach achieves higher performance than the existing methods. Moreover, comparative visual results show the effectiveness of the proposed method in restoring saturated information from original input images and mitigating ghosting artifacts caused by large movement of objects. Ablation studies show the effectiveness of the proposed method, architectural choices, and modules for efficient HDR reconstruction.

Keywords: high-dynamic-range imaging; spatial attention; non-local means; deep learning

check for updates

Citation: Yoon, H.; Uddin, S.M.N.; Jung, Y.J. Multi-Scale Attention-Guided Non-Local Network for HDR Image Reconstruction. *Sensors* **2022**, *22*, 7044. https://doi.org/10.3390/s22187044

Received: 21 July 2022
Accepted: 4 September 2022
Published: 17 September 2022

Publisher's Note: MDPI stays neutral with regard to jurisdictional claims in published maps and institutional affiliations.

Copyright: © 2022 by the authors. Licensee MDPI, Basel, Switzerland. This article is an open access article distributed under the terms and conditions of the Creative Commons Attribution (CC BY) license (https://creativecommons.org/licenses/by/4.0/).

1. Introduction

This supplementary file accompanies the manuscript "Multi-scale Attention-Guided Non-local Network for HDR Image Reconstruction" and contains the architecture of the proposed method.

Table S1. Architecture of the Encoder

Name	Input	Output (channel)	Type
Input	6		3 Input images
Double Conv-Layer	6	64	(Conv 3 × 3 + ReLU) ×2
Double Conv-Layer	64	128	(Conv 3 × 3 + ReLU) ×2
Spatial attention input (first scale)	64		Input Feature Maps
Conv	64	128	Conv 3 × 3
ReLU	128	128	ReLU Activation
Conv	128	64	Conv 3 × 3
Sigmoid	64	64	Sigmoid Activation
Conv	192	64	Conv 3 × 3
Spatial attention input (Second scale)	128		Input Feature Maps
Conv	128	256	Conv 3 × 3
ReLU	256	256	ReLU Activation
Conv	256	256	Conv 3 × 3
Sigmoid	128	128	Sigmoid Activation
Conv	384	128	Conv 3 × 3
Double Conv-Layer	192	128	(Conv 3 × 3 + ReLU) ×2

Table S2. Architecure of the Decoder

Name	Input	Output (channel)	Type
RDB input	128		Input Feature Maps
Conv-Layer	128	32	Conv 3 × 3 + ReLU
Conv-Layer	160	32	Conv 3 × 3 + ReLU
Conv-Layer	192	32	Conv 3 × 3 + ReLU
Conv-Layer	224	32	Conv 3 × 3 + ReLU
Conv-Layer	256	32	Conv 3 × 3 + ReLU
Conv-Layer	288	128	Conv 1 × 1 + ReLU
APNB input	128		Input Feature Maps
Conv-Layer	128	64	Conv 1 × 1 + ReLU
Conv-Layer	128	64	Conv 1 × 1 + ReLU
Conv-Layer	64	128	Conv 1 × 1 + ReLU
Conv-Layer	128	64	Conv 3 × 3 + ReLU
Conv-Layer	64	64	Conv 3 × 3 + ReLU
Conv-Layer	64	3	Conv 1 × 1 + ReLU
Output	3		Output image
